# Supplementary material for: Human GPR174 deficiency drives polyclonal lymphoproliferative disease via defects in T cell function
Source: medRxiv. 2026 Jul 17:2026.07.14.26357774. Preprint. [Version 1] doi: 10.64898/2026.07.14.26357774 (PMC13409280; doi:10.64898/2026.07.14.26357774)

Supplementary Figure 1

A

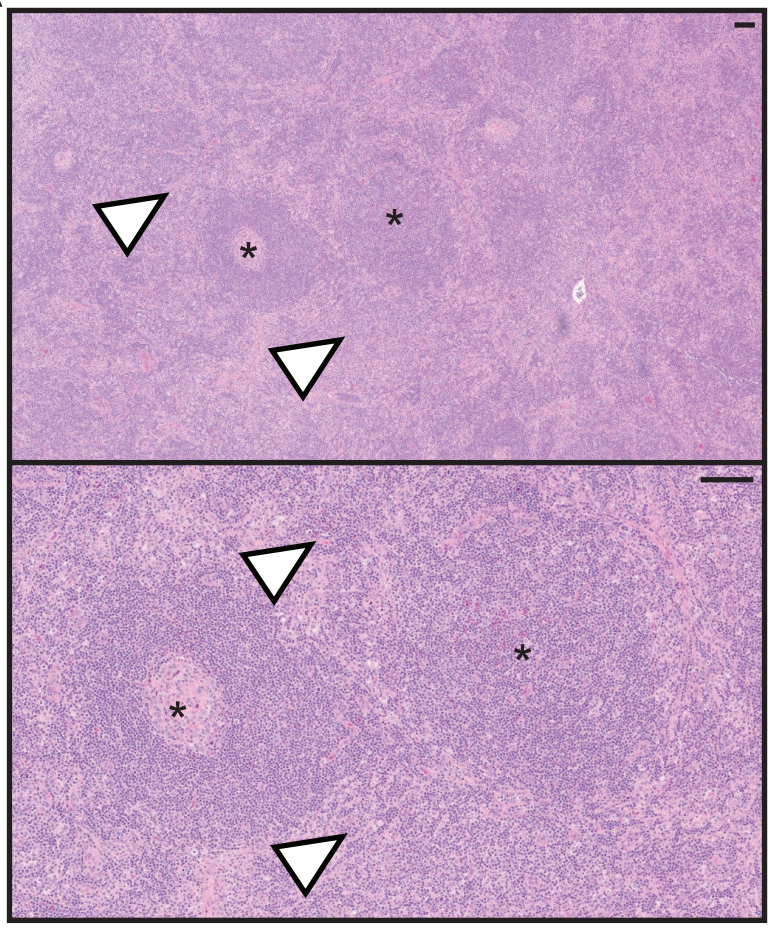

B

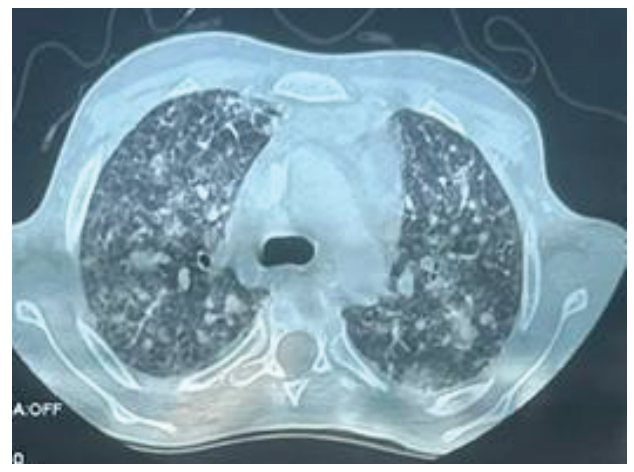

Supplement: Supplementary file 1 [file media-1.pdf]
